# Is Sleeve Gastrectomy as Effective in Older Patients as in Younger Patients? A Comparative Analysis of Weight Loss, Related Comorbidities, and Medication Requirements

**DOI:** 10.1007/s11695-022-05940-1

**Published:** 2022-04-12

**Authors:** Sonia Fernández-Ananín, Eulalia Ballester, Berta Gonzalo, Claudia Codina, Inka Miñambres, Antonio Pérez, Ignasi J. Gich, Sandra González, Cristina Serrano, Carmen Balagué

**Affiliations:** 1 Universitat Autònoma de Barcelona (UAB), Medical School, Department of General and Digestive Surgery, Hospital de La Santa Creu i Sant Pau, Barcelona, Spain; 2 Functional Unit of Bariatric & Metabolic Surgery, Hospital de La Santa Creu I Sant Pau, Universitat Autònoma de Barcelona (UAB), Medical School, Barcelona, Spain; 3 Department of Endocrinology and Nutrition, CIBER of Diabetes and Metabolic Diseases (CIBERDEM), Hospital de La Santa Creu I Sant Pau, Universitat Autònoma de Barcelona (UAB), Medical School, Barcelona, Spain; 4 Department of Epidemiology, Hospital de La Santa Creu I Sant Pau, Universitat Autònoma de Barcelona (UAB), Medical School, Barcelona, Spain; 5 Department of Dietetics, Hospital de La Santa Creu I Sant Pau, Universitat Autònoma de Barcelona (UAB), Medical School, Barcelona, Spain

**Keywords:** Bariatric surgery, Morbid obesity, Metabolic surgery, Sleeve gastrectomy, Older patients, Comorbidities

## Abstract

**Background:**

Bariatric surgery in the older population has been the subject of ongoing debate but several studies have recently demonstrated its short-term advantages in this age group. It is not yet clear, however, whether these benefits are long-lasting.

**Methods:**

We retrospectively analyzed patients with morbid obesity who underwent laparoscopy sleeve gastrectomy (LSG). These patients were divided into two groups: those above 60 years of age (older group) and those of 60 years or under (younger group). Variables evaluated included demographics and anthropometrics data, comorbidities, and daily medication requirements.

**Results:**

Two hundred fifty-two patients underwent LSG, 57 in the older group and 195 in the younger group. Outcomes related to weight loss in the older subjects were modest compared to those in the younger population (older group %EWL 41.6 vs younger group %EWL 51.1, *p* < 0.05, older group %TWL 24.9% vs younger group %TWL 25.2%, *p* < 0.05). During follow-up, both older and younger patients showed an improvement in all the comorbidities: hypertension (older 82.5% vs 38.1%, younger 52.6% vs 29.2%, *p* < 0.05), type 2 diabetes mellitus (older 38.6% vs 27.3%, 34.9% vs 23.9%, *p* < 0.05), hyperlipidemia (older 75.4% vs 42.9%, younger 35.9% vs 21.1%, *p* < 0.05), and OSAHS (older 57.9% vs 30%, younger 40.4% vs 7.1%, *p* < 0.05). The average number of daily medications used to manage comorbidities decreased in both groups.

**Conclusion:**

LSG in older patients is effective in terms of weight loss, improvement of comorbidities, and lower daily medication requirements up to 5 years of follow-up.

**Graphical abstract:**

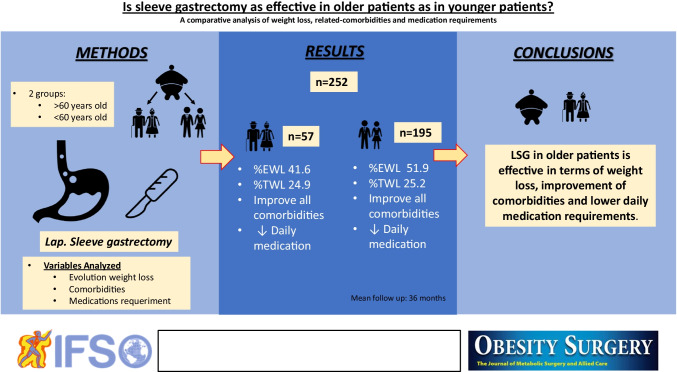

## Introduction

The prevalence of obesity in the older population is on the rise in pace with the ageing population (https://www.who.int/news-room/fact-sheets/detail/obesity-and-overweight) [1]. With advancing age, obesity is associated with many comorbidities, such as hypertension, diabetes, arthritis, cardiovascular disease, and reduced quality of life [2]. The most effective and long-lasting treatment for obesity and its coexisting diseases is bariatric surgery [3, 4]. Traditionally however, older obese patients were not referred for bariatric surgery as the risks were considered high. In addition, initial studies showed poor results regarding weight loss and the number of postoperative complications was higher than in younger subjects [5–10]. Current scientific evidence, nevertheless, supports the beneficial effects of bariatric surgery in older patients. It has shown to improve comorbidities and reduce daily medication requirements, and morbidity and mortality are acceptable [11–23]. The numbers of patients in studies carried out to date are low, however, and the follow-up period is short.

In a study published in 2014 [24], we found that although weight loss at 1-year follow-up was lower in the older group than in the younger group, the improvement or remission of comorbidities and the reduction in daily drugs supported the use of this surgical procedure in older patients.

The objective of this study was to carry out a comparative evaluation of the results in the medium and long term between the group of young and elderly patients.

## Material and Methods

### Population and Study Design

Between January 2008 and February 2019, we prospectively collected data from our database of patients undergoing LSG at our institution. We divided patients into two groups, those over 60 years and those of 60 years or under. The cut-off age of 60 years was based on the definition of the United Nation for the older population (https://www.un.org/en/sections/issues-depth/ageing/).

Variables analyzed included patients’ demographic data, preoperative weight and metrics, preoperative comorbidities related to obesity (type 2 diabetes mellitus (T2DM), hypertension, hyperlipidemia, and obstructive sleep apnea/hypopnea syndrome (OSAHS)), the need for daily medication, and global surgical outcomes. We assessed the evolution of these parameters up to 5 years after surgery.

The primary endpoints of the study were to analyze the mid-to-long-term impact of bariatric surgery on improving and resolving comorbidities in the older group compared to the younger group and to assess long-term daily medication requirements. The secondary outcome was the evolution of weight loss during the same period.

The study was conducted according to STROBE guidelines [25]. All patients were discussed at a multidisciplinary metabolic and bariatric committee prior to surgery and the criteria used for LSG were based on the Interdisciplinary European Guidelines on Metabolic and Bariatric Surgery [26]. The study was approved by the local ethics committee and all patients signed the informed consent form. The study was performed in compliance with the Declaration of Helsinki principles for medical research.

### Surgical Technique

We performed a standard LSG, using a 36 Fr size bougie. Staple lines were reinforced with a synthetic buttressing material (GORE® SEAMGUARD®). Methylene blue test was used to evaluate the integrity of the staple line. Drainage was not routinely left. Patients started a liquid diet on the first postoperative day. They were discharged on the second day after LSG if evolution was favorable.

The Clavien-Dindo classification was used to define postoperative complications that occurred within the first 30 days after surgery [27].

### Outcomes Assessed

#### Comorbidities Analyzed

We focused on the evolution of obesity-related comorbidities: hypertension, T2DM, hyperlipidemia, and OSAHS.

Remission of hypertension was defined as blood pressure of at least 140/90 mmHg with cessation of antihypertensive drugs by the primary care physician. The criteria for remission of type 2 diabetes followed the current American Diabetes Association recommendations. Remission of hyperlipidemia was defined as LDL cholesterol levels below 160 mg/dl and triglycerides below 200 mg/dl without medication. Remission of OSAHS was defined as the discontinued need for continuous positive airway pressure (CPAP) confirmed by polysomnography [28–31].

#### Weight Loss Outcomes

BMI, %EWL, and %TWL were used to report the results of weight loss endpoints [32]. Metrics data were extracted in both groups at three time points: at 1, 3, and 5 years after LSG.

Success rates were analyzed according to Reinhold modified by Christou [33] and Biron criteria [34].

#### Medication Requirements

Before and after surgery, we recorded the number of daily medications to control obesity-related comorbidities and the need for CPAP.

### Follow-Up

Follow-up was performed by specialists in bariatric surgery, endocrinology, and dietetics. Each of the comorbidities was evaluated before surgery and during follow-up by the corresponding specialist. Surgical follow-up was performed on the 7th postoperative day, at 3 and 12 months after surgery, and then annually until 5 years after surgery.

### Statistical Analysis

Data were assessed using the chi-square test for categorical variables and the *T*-test for continuous variables. Data for continuous variables were expressed as the mean difference (MD) and 95% confidence interval (CI) and data for dichotomous variables as percentage. To determine the influence of two different categorical independent variables on one continuous dependent data and their interaction, we applied two-way analysis of variance (ANOVA).

A *p* value < 0.05 was considered statistically significant for all the analysis. The IBM-SPSS® 26.0 (SPSS, Inc., Chicago, IL, USA) software platform was used for statistical analysis.

## Results

### Baseline Characteristics of the Cohorts

A total of 252 patients were submitted to LSG during the study period, 57 (22.6%) in the older group (> 60 years of age) and 195 (77.3%) in the younger group (≤ 60 years of age). The mean age of the cohort was 51.5 years (range 21–68). The mean age of the older patients was 63 years (range 61–68) and mean age of the younger patients was 48 years (range 21–60). Most patients in both groups were women, 46 (80.7%) vs 11 men (19.3%) in the older group and 137 (70.3%) vs 58 men (29.7%) in the younger group (*p* = 0.13). Table 1 shows the demographic data for the subgroups and for the whole sample.

**Table 1:** Demographic and anthropometric characteristics

	Total *n* = 252	> 60 *n* = 57 (22.6%)	≤ 60 *n* = 195 (77.3%)	*p*
Age (years) Mean (range)	51.5 (21–68)	63 (61–68)	48 (21–60)	
Gender Female, *n* (%) Male, *n* (%)	183 (72.6%) 59 (27.3%)	46 (80.7%) 11 (19.3%)	137 (70.3%) 58 (29.7%)	*p* > 0.05
Weight (kg)	119.9 ± 23.5	113.6 ± 17.5	121.8 ± 24.7	*p* < 0.05
BMI (kg/m^2^)	44.6 ± 6.9	44.4 ± 7.2	44.6 kg ± 6.2	*p* > 0.05
Overweight (kg)	61.6 ± 19.8	58.6 ± 15.6	62.5 ± 20.8	*p* > 0.05

Abbreviations: *BMI*, body mass index

^*^Values expressed as mean ± SD

The mean preoperative weight was higher in the younger group (121.8 ± 24.7 vs 113.6 ± 17, *p* < 0.05), reaching statistical significance. No significant differences were observed between the two groups regarding initial BMI (younger group 44.6 ± 7.2 vs older group 44.4 ± 6.2, *p* > 0.05) or for preoperative overweight (younger group 62.5 ± 20.8 vs older group 58.6 ± 15.6, *p* > 0.05).

One or more obesity-related comorbidities were present in 66.5% (*n* = 167) of the cohort: 82.5% (*n* = 47) in the older group and 61.9% (*n* = 120) in the younger group. The difference was statistically significant (*p* = 0.04). Preoperative obesity-related comorbidities were significantly higher in the older group (hypertension 82.5% vs 52.6%, *p* < 0.05; T2DM 38.6% vs 34.9%, *p* > 0.05; hyperlipidemia 75.4% vs 35.9%, *p* < 0.05; sleep apnea 57.9% vs 40.4%, *p* < 0.05) (see Table 2).

**Table 2:** Preoperative comorbidities

	Total	> 60	≤ 60	*p*
No comorbidities *n* (%)	84 (33.5%)	10 (17.5%)	74 (38.1%)	*p* < 0.05
≥ 1 comorbidities	167 (65.5%)	47 (82.5%)	120 (61.9%)	*p* < 0.05
Hypertension	149 (59.4%)	47 (82.5%)	102 (52.6%)	*p* < 0.05
Type 2 DM	83 (32.9%)	22 (38.6%)	61 (31.3%)	*p* > 0.05
Hyperlipidemia	113 (44.8%)	43 (75.4%)	70 (35.9%)	*p* < 0.05
OSAS treatment with CPAP	111 (44.4%)	33 (57.95)	78 (40.4%)	*p* < 0.05

Obesity-related comorbidities analyzed*

Abbreviations: *OSAS*, obstructive sleep apnea syndrome

### Surgical Outcomes and Early Complications

All procedures were performed laparoscopically and in all cases, LSG was the first bariatric procedure the patients had undergone. Total mean operative time was 102.3 min and there were no differences between groups (younger group 102.7 min vs elderly group 101.2 min, *p* > 0.05). Median length of hospital stay was 2.72 days in the younger group and 3.03 days in the older group, with no statistically significant differences. There were no conversions to open surgery in either group.

Overall postoperative complications were similar in both groups. In the total cohort, 3 patients required surgical intervention under general anesthesia due to staple line leak (2 patients in the younger group and 1 patient in the older group). One patient from the younger group required admission to intensive care. No mortality occurred in either group in the first 90 days after surgery.

Immediate postoperative complications according to Dindo-Clavien’s [27] classification are detailed in Table 3.

**Table 3:** Surgical outcomes and postoperative complication (30-day post-surgery)

	Total	> 60	≤ 60	*p*
Operating time (min), mean	102.38 ± 36.4	101.2 ± 33.0	102.7 ± 34.4	*p* > 0.05
Length of hospital stay, (day) mean	2.96 ± 4.3	3.03 ± 1.4	2.72 ± 4.8	*p* > 0.05
Conversion to open	0	0	0	*p* > 0.05
Re-intervention	5 (2%)	1 (1.8%)	4 (2.1%)	*p* > 0.05
No surgical complications	242 (96%)	56 (99.5%)	186 (96%)	*p* > 0.05
Clavien-Dindo classification (29) I II III IV V	2 (0.8%) 5 (2.0%) 2 (0.8%) 2 (0.8%) 1 (0.4%) 0 (0%)	0 (0%) 0 (0%) 1 (0.5%) 1 (0.5%) 0 (0%) 0 (0%)	2 (1%) 5 (2.0%) 1 (0.5%) 1 (0.5%) 1 (0.5%) 0 (0%)	*p* > 0.05

### Follow-Up

The average follow-up after surgery was 33 ± 21.8 months and the median was 36 months (range 6–60). From the total group, 34.1% (*n* = 86) fulfilled the follow-up at 5 years after surgery. Of this subgroup, 64 were from the younger group and 22 from the older group. Thirty percent of the cohort was lost to follow-up. Patient loss was lower in the older group than in the younger participants but the difference was not statistically significant.

### Resolution of Comorbidities

The evolution of comorbidities was favorable in both groups during follow-up after LSG. In the older subjects, hypertension dropped from 82.5 to 38.1% in up to 5 years, T2DM from 38.6 to 27.3%, hyperlipidemia from 75.4 to 42.9%, and OSAHS from 57.9 to 30%. In the younger patients, hypertension improved from 52.6 to 29.2% in up to 5 years of follow-up, T2DM from 34.9 to 23.9%, hyperlipidemia from 35.9 to 21.1%, and OSAHS from 40.4 to 7.1%.

At a mean follow-up of 33 months, the comorbidities that showed the highest percentage of improvement in older patients were hypertension (53.8%), OSAHS (48.1%), hyperlipidemia (43.1%), and T2DM (29.9%). In contrast, in the younger patients, the order of improvement was OSAHS (82.4%), hypertension (44.4%), hyperlipidemia (41.2%), and T2DM (31.5%).

The improvement in comorbidities was similar to that observed at 33 months. Hypertension decreased by 32.8% in the younger group (*p* = 0.001) and by 36.4% in the older group (*p* = 0.008). OSAHS dropped 31.1% in the younger group and 40.9% in the older group, hyperlipidemia dropped 19.7% in the younger group (*p* = 0.13) and 36.4% in the older group (*p* = 0.39), and T2DM improved by 19.4% in the younger patients (*p* < 0.05) and by 18.2% in the older group (*p* > 0.05).

### Weight Loss Evolution

**Fig. 1:**
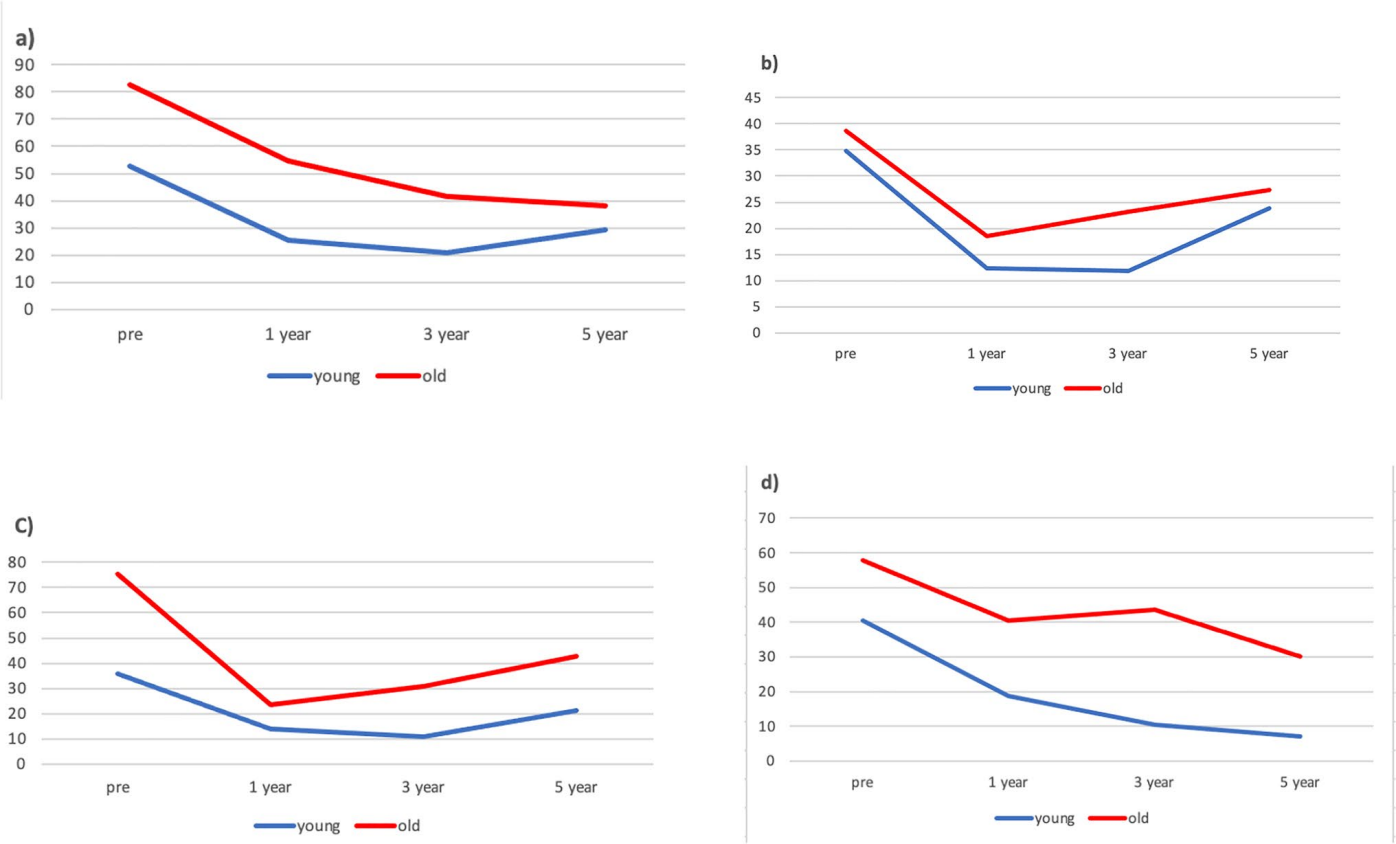
Evolution of comorbidities in both groups after surgery during the study period. **a**) Hypertension ; **b**) T2DM ; **c**) Hyperlipidemia; **d**) OSAHS

Mean preoperative BMI was similar in both groups (younger group: 44.3 kg/m^2^, older group: 43.2 kg/m^2^) and BMI also decreased in both groups during the 5 years after LSG. In the ≤ 60 years group, mean BMI in the 1st postoperative year was 30.3 kg/m^2^ and 32.7 kg/m^2^ in the 5th year after LSG. In the > 60 years patients, mean BMI was 31.2 to 33.7 kg/m^2^ at 5 years after surgery (Fig. 1).

**Fig. 2:**
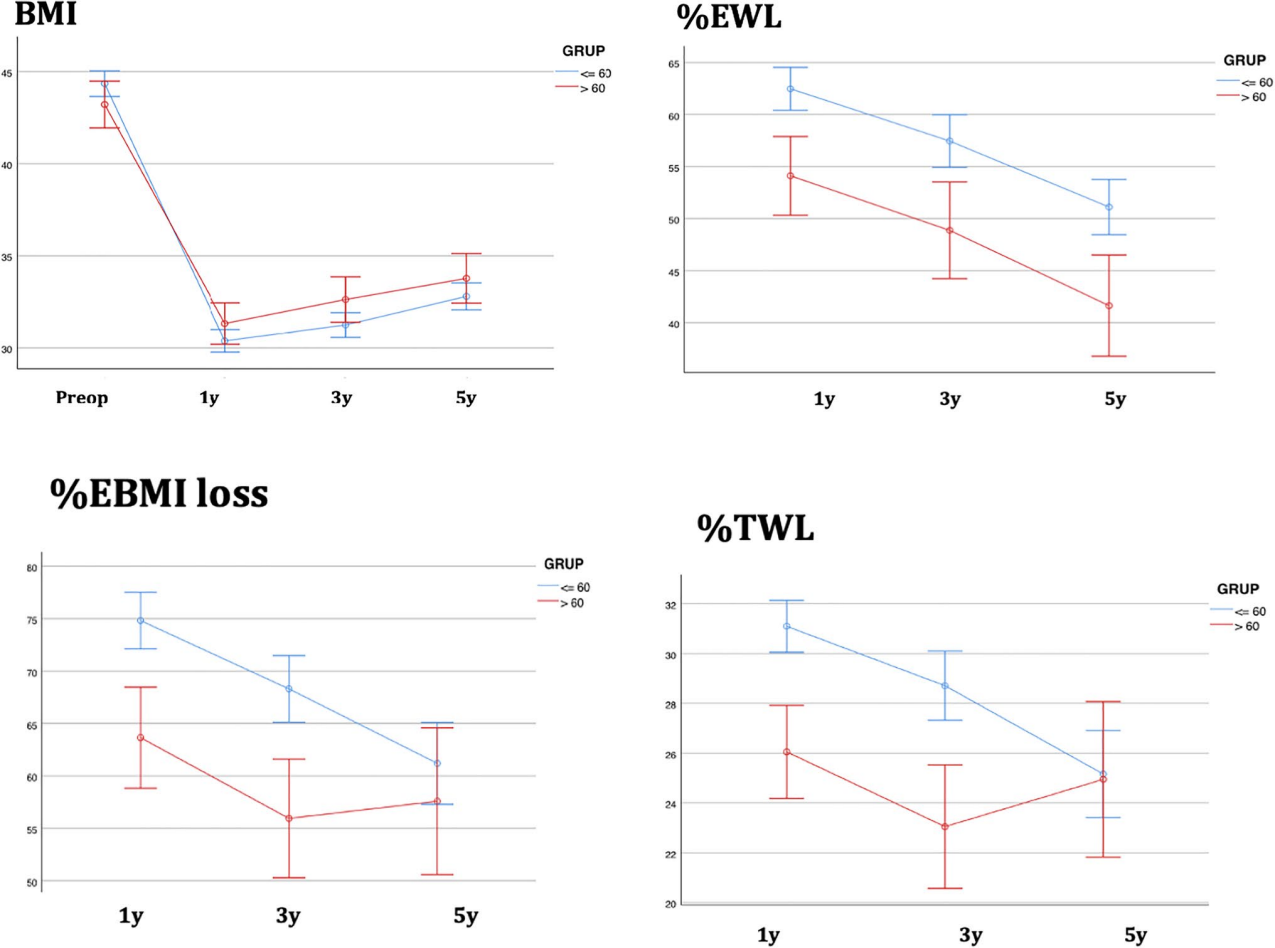
Evolution of anthropometric parameters (BMI, %EWL, %TWL, %EBMI loss) in both groups after surgery during the study period

In a period of up to 5 years after LSG, the total cohort presented an %EWL of 48.2 ± 21.7, which is classified as good according to Reinhold’s criteria. Assessment by group showed the younger group had significantly better results than the older group (51.1% vs 41.6% *p* < 0.05). The total cohort showed a %TWL of 24.7 ± 13.9 (Table 4 and Fig. 2). The percentages of these quality indicators evolved in a similar pattern in both groups. Older patients had a significantly lower %TWL than the younger patients (24.9 vs 25.2 *p* < 0.05) (see Table 4).

**Table 4:** Anthropometric outcomes and their evolution after sleeve gastrectomy

	Baseline	1st year after LSG	3rd year after LSG	5th year after LSG
*n* (%) Global > 60 ≤ 60	252 57 195	80 (31.7%) 15 (26.2%) 65 (33.3%)	56 (22.2%) 16 (28.0%) 40 (20.5%)	86 (34.1%) 20 (35.0%) 66 (33.8%)
BMI (kg/m^2^) Global > 60 ≤ 60	44.6 ± 6.9 43.2 44.3	31.3 ± 6.1 31.3 30.3	32.6 ± 6.4 32.6 31.2	33.2 ± 5.9 33.7 32.7
%EWL Global > 60 ≤ 60		59.1 ± 18.9 54.1 62.4	52.5 ± 20.5 48.8 57.4	48.2 ± 21.7 41.6 51.1
%TWL Global > 60 ≤ 60		29.5 ± 9.1 26.0 31.1	26.57 ± 10.5 23.0 28.7	24.7 ± 13.9 24.9 25.2

Values are expressed as mean ± SD

### Changes in Daily Medication Requirements

The number of medications used to treat these specific conditions was recorded twice: first, before surgery and again at follow-up, at up to 5 years after surgery. The mean number of drugs required to treat comorbidities decreased in both groups after surgery and remained lower than the initial numbers at the most recent follow-up.

In the older group, the average number of medications used to treat these comorbidities was 2.4 ± 1.5 per day before surgery and 1.1 ± 1.3 during the follow-up (*p* = 0.02). In the younger group, medication requirements dropped from 1.3 ± 1.2 to 0.6 ± 1 (*p* = 0.02).

Of the 86 patients who completed a 5-year follow-up, the mean pharmacological requirements to treat obesity-related comorbidities decreased significantly in both age groups (younger group *n* = 64, 1.4 + / − 1.3 to 0.7 + / − 1.1, *p* < 0.001; older group *n* = 22, 2.8 + / − 1.9 to 1.4 + / − 1.6, *p* < 0.001).

## Discussion

This study assesses the long-term evolution of comorbidities after bariatric surgery in patients over 60 years old. Our findings show that the improvement or resolution of obesity-related conditions in the older population persisted during up to 5 years of follow-up after LSG. We also found that daily medication requirements to control these comorbidities decreased in the long-term.

In both cohorts, all the comorbidities studied improved after bariatric surgery and this improvement remained throughout the follow-up. On examining each comorbidity individually, T2DM and hyperlipidemia improved until the third year in both populations. This improvement tapered off thereafter but did not return to preoperative levels in either group. Hypertension evolved differently in the two groups. While in older patients, the improvement was progressive for up to 5 years, in young subjects, the greatest decrease was appreciated in the 3rd year after surgery, after which it increased slightly. However, OSAHS continued to improve until the end of the follow-up in both groups.

The long-term findings regarding T2DM in our cohort over 60 years are encouraging. The improvement in type 2 diabetes mellitus at 5 years was similar to that in the younger group. Medical evidence to date suggests that the percentage of remission of diabetes after obesity surgery varies considerably depending on factors such as the type of bariatric procedure, the duration of the disease, and metabolic control [35–38]. Results are generally better in patients with a recent onset of diabetes and greater weight loss, which could account for the modest improvement in this comorbidity in older patients.

However, although diabetes was not the comorbidity that showed the best results, the findings in our elderly cohort, with an improvement of almost 30% regarding the baseline incidence, reinforce the indication and the benefits of bariatric surgery in this population group.

The weight loss achieved in the older patients merits discussion. In a follow-up of up to 5 years, the total cohort presented an %EWL of 48.2 ± 21.7%, which is classified as good according to Reinhold’s criteria. However, when we compare our two groups, the younger patients had significantly better results in terms of weight loss than the older group (51.1 vs 41.6%, respectively, *p* < 0.05). These findings agree with those presented by other authors such as Mizrahi et al. (%EWL 75 vs 62%, *p* = 0.001) [39], Van Rutte et al. (%EWL 75 vs 62, *p* = 0.001) [12], Al-Kurd et al. (%EWL 72.9 vs 63.7%, *p* = 0.001) [15], and Kaplan et al. (%EWL 60.7 vs 56.3%, *p* = 0.001) [40]. These authors showed better results in their older cohort than ours, but their respective follow-ups were shorter (12, 14.6, 31.3, and 12 months, respectively).

A higher prevalence of comorbidities in older patients than in their younger counterparts may be a reason for bias related to serious postoperative complications. However, our findings reinforce the safety of LSG in older patients. Evidence from our series and other similar studies is that 30-day surgical complications are low and comparable between younger and older patients. Our data support findings of authors such as Willkomm et al. [20], Van Rutte et al. [12], and Navarrete et al. [19], who also reported a mortality rate of 0%. Furthermore, in a recent meta-analysis published by Vallois et al. [41], mortality after bariatric surgery in both younger and older groups was 0.2%.

The low morbidity and mortality rate of bariatric surgery in this population group today has several explanations. The increasing popularity of bariatric techniques, surgeon’s experience and standardization of minimal invasive surgery, the overcoming of the learning curve, and the application of prehabilitation programs before surgery have all contributed to the implementation of these procedures in older patients [42].

The main limitation of our study is the retrospective nature of the data collection and its consequent inferior level of evidence compared to prospective studies. The main strength is the monitoring of the cohort for up to 5 years, which is uncommon in studies of this type of publication. According to the expert consensus review [43], one of the main limiting factors for obtaining robust findings is the loss of patients during the follow-up. To consider a follow-up as adequate, it should follow at least 60% of the cohort for a minimum of 5 years after surgery. Most bariatric surgery studies to date followed patients for less than 2 years, although Navarrete et al. [19] recently analyzed results in their cohort at 3 years of follow-up. We followed 70% of our total cohort for 5 years.

Another strong point of our study is the large sample size compared to previous similar articles. Our study included 57 older patients, while others included 3 patients (Leivonen et al. [44]), 12 (Burchett et al. [45]), 7 (Abbas et al. [46]), and 18 (González-Heredia et al. [47]).

Finally, we wish to emphasize that the main goal of bariatric surgery in the elderly is to resolve or improve obesity-related comorbidities, not only in order to enhance quality of life but also to increase survival. Further research into this topic, however, has yet to be carried out. Both our study and the other studies published to date focus only on comorbidities directly related to morbid obesity. The analysis of other comorbidities, such as those involving the locomotor system or psychiatric issues, might also reveal positive findings. It might also be kept in mind that older patients likely have lower concern for body image, in which case resolution of comorbidities and the consequent reduction of daily drug requirements can translate into a higher score on the quality of life test. Future studies might consider such aspects.

## Conclusion

Laparoscopic sleeve gastrectomy in the older population can provide a long-term improvement in comorbidities such as T2DM, hypertension, hyperlipidemia, and sleep apnea, and decrease daily medication requirements. This surgical approach to obesity has proven to be a safe technique in older patients, with surgical complications being similar to those in their younger counterparts.

## Abbreviations

LSG: Laparoscopic sleeve gastrectomy
LRYGB: Laparoscopic Roux-en-Y gastric bypass
BMI: Body mass index
T2DM: Type 2 diabetes mellitus
OSAHS: Obstructive sleep apnea/hypopnea syndrome
CPAP: Continuous positive airway pressure
%EWL: Percent excess body weight percentage
%TWL: Percent total weight loss
